# Applied comparison of large‐scale propensity score matching and cardinality matching for causal inference in observational research

**DOI:** 10.1186/s12874-021-01282-1

**Published:** 2021-05-24

**Authors:** Stephen P. Fortin, Stephen S Johnston, Martijn J Schuemie

**Affiliations:** 1 Janssen R&D, LLC, Raritan, NJ USA; 2 Johnson & Johnson, New Brunswick, NJ USA

**Keywords:** Cardinality matching, Propensity score matching, Causal inference, Residual bias, Systematic error, Sample size, Balance, Hypertension, ACEI, Diuretic

## Abstract

**Background:**

Cardinality matching (CM), a novel matching technique, finds the largest matched sample meeting prespecified balance criteria thereby overcoming limitations of propensity score matching (PSM) associated with limited covariate overlap, which are especially pronounced in studies with small sample sizes. The current study proposes a framework for large-scale CM (LS-CM); and compares large-scale PSM (LS-PSM) and LS-CM in terms of post-match sample size, covariate balance and residual confounding at progressively smaller sample sizes.

**Methods:**

Evaluation of LS-PSM and LS-CM within a comparative cohort study of new users of angiotensin-converting enzyme inhibitor (ACEI) and thiazide or thiazide-like diuretic monotherapy identified from a U.S. insurance claims database. Candidate covariates included patient demographics, and all observed prior conditions, drug exposures and procedures. Propensity scores were calculated using LASSO regression, and candidate covariates with non-zero beta coefficients in the propensity model were defined as matching covariates for use in LS-CM. One-to-one matching was performed using progressively tighter parameter settings. Covariate balance was assessed using standardized mean differences. Hazard ratios for negative control outcomes perceived as unassociated with treatment (i.e., true hazard ratio of 1) were estimated using unconditional Cox models. Residual confounding was assessed using the expected systematic error of the empirical null distribution of negative control effect estimates compared to the ground truth. To simulate diverse research conditions, analyses were repeated within 10 %, 1 and 0.5 % subsample groups with increasingly limited covariate overlap.

**Results:**

A total of 172,117 patients (ACEI: 129,078; thiazide: 43,039) met the study criteria. As compared to LS-PSM, LS-CM was associated with increased sample retention. Although LS-PSM achieved balance across all matching covariates within the full study population, substantial matching covariate imbalance was observed within the 1 and 0.5 % subsample groups. Meanwhile, LS-CM achieved matching covariate balance across all analyses. LS-PSM was associated with better candidate covariate balance within the full study population. Otherwise, both matching techniques achieved comparable candidate covariate balance and expected systematic error.

**Conclusions:**

LS-CM found the largest matched sample meeting prespecified balance criteria while achieving comparable candidate covariate balance and residual confounding. We recommend LS-CM as an alternative to LS-PSM in studies with small sample sizes or limited covariate overlap.

**Supplementary Information:**

The online version contains supplementary material available at 10.1186/s12874-021-01282-1.

## Background

Randomization tends to produce comparable study groups in terms of both observed and unobserved covariates in controlled experimentation. Unfortunately, random assignment of treatment is conspicuously absent from observational studies [1]. In the absence of randomization, differences in covariate distributions between study groups may prevent valid statistical inference from data [2]. As such, a key component in the design of observational studies includes addressing the presence of confounding covariates to reduce study bias using statistical methods such as matching [3–5].

While propensity score matching (PSM) is the most ubiquitous matching technique for causal inference in observational research, the technique is subject to limitations. First, PSM is susceptible to substantial bias, large variance in estimates and poor sample retention in studies with limited overlap of covariate distributions between study groups [4, 6, 7]. Second, due to limited degrees of freedom, restrictions on the number of matching covariates used may be necessary to avoid model over-parameterization and overfitting although this may be overcome through large-scale propensity score matching using machine learning to calculate propensity scores [8, 9]. These limitations are especially pronounced in studies with small sample sizes.

A novel matching method, cardinality matching (CM), uses recent advancements in integer programming to find the largest matched sample meeting a set of prespecified balance criteria [4]. For instance, CM solves for the optimal (i.e., largest) matched sample subject to investigator-defined constraints on the maximum standardized mean difference of covariates between study groups. By matching directly on the original covariates rather than propensity scores, CM handles issues of limited overlap of covariate distributions and maximizes sample size retention while meeting covariate balance criteria [4].

Prior literature comparing PSM and CM is primarily focused upon measures of post-match sample retention and matching covariate balance [4, 10]. Nevertheless, residual bias may arise due to confounding from unobserved variables or unadjusted covariate candidates [11, 12]. Large-scale matching on high-dimensional datasets may indirectly adjust covariate candidate balance and reduce residual confounding, which may be measured through candidate covariate balance and negative control outcome experiments, respectively, due to the complex interactions between covariates in real-world healthcare data. Specifically, negative control outcome experiments estimate the systematic error and, therefore, residual bias unattributable to random chance based on negative control outcomes perceived as unassociated with treatment [11].

The current study proposes a framework for the empirical selection of matching covariates in large-scale CM (LS-CM); and compares the performance of large-scale PSM (LS-PSM) and LS-CM in an observational study of new users of angiotensin converting enzyme inhibitor (ACEI) vs. thiazide or thiazide-like diuretic monotherapy. To simulate a diverse variety of potential research conditions, both matching techniques are evaluated in terms of post-match sample size, matching and candidate covariate balance and residual bias at progressively smaller sample sizes possessing increasingly limited covariate overlap.

## Methods

### Study design and data source

We conducted a retrospective comparative new-user cohort study in the IBM® MarketScan® Commercial Claims and Encounters Database (CCAE), which primarily consists of de-identified, patient-level health data from over 142 million individuals enrolled in employer-sponsored health insurance plans in the United States. The CCAE database includes adjudicated health insurance claims (inpatient, outpatient, and prescription) and enrollment data from large employers and health plans who provide private insurance coverage. Data were standardized to the Observational Health and Data Sciences and Informatics (OHDSI) Observational Medical Outcomes Partnership (OMOP) Common Data Model (CDM) version 5.3, which maps international coding systems into standard vocabulary concepts [13]. In the United States, retrospective analyses of the CCAE data are considered exempt from informed consent and institutional review board (IRB) approval as dictated by Title 45 Code of Federal Regulations, Part 46 of the United States, specifically 45 CFR 46.104 (d)(4).

### Study population

We identified new users of ACEI and thiazide or thiazide-like diuretic monotherapy between October 1, 2014 and January 1, 2017. For each patient, we defined the *index* as the date of first drug exposure.

The study was limited to patients with a minimum of 365 days of continuous observation in the database prior to index. We required patients to have a recorded diagnosis for hypertension at or within 365 days prior to index (see Supplemental Appendix A for a list of codes used to query the database). As described in Suchard et al., new users were defined as patients whose first observed treatment for hypertension was ACEI or thiazide or thiazide-like diuretic monotherapy [14]. Patients with exposure to any other active ingredient listed within the five primary drug classes for the treatment of hypertension in the 2017 American College of Cardiology/American Heart Association (ACC/AHA) guidelines (i.e., ACEI, thiazide or thiazide-like diuretics, angiotensin receptor blockers, dihydropyridine calcium channel blockers, non- dihydropyridine calcium channel blockers) any time prior to or within 7 days post-index were excluded [14, 15]. Patient inclusion criteria and attrition are summarized in Supplemental Appendix B.

### Sample groups

We developed a total of four progressively smaller sample groups, including a full study population group and a 10 %, 1 and 0.5 % subsample group. Analyses were performed across each aforementioned sample group to simulate a diverse set of research conditions, specifically permitting comparison of LS-PSM and LS-CM in the setting of increasingly limited covariate overlap. The scarcity of negative control outcomes observed at smaller sample sizes necessitated pooled analyses of negative control outcome experiments across multiple subsample draws within each subsample group. As such, the 10 %, 1 and 0.5 % subsample groups included 5, 50 and 100 subsample draws, respectively. Each subsample draw was performed by random sampling without replacement from the study population stratified by study comparison group.

### Patient demographic and clinical characteristics

We measured patient demographics at index including age, grouped into categories in 5-year increments; sex; and index year and month. Patient clinical characteristics included all observed condition, drug exposure, measurement and observation codes occurring within a long-term or short-term window (i.e., at or within 365 or 30 days prior to index, respectively) with the exception of ingredient-level drug exposures to ACEIs and thiazide or thiazide-like diuretics. All drug exposures were grouped at both the ingredient-level and according to the Anatomical Therapeutic Chemical (ATC) classification system. Patient comorbidities were measured using the Charlson Comorbidity Index (CCI) [16]. Finally, we measured the following disease severity and risk scores: Diabetes Complications Severity Index (DCSI), CHADS_2_ score, and CHA_2_DS_2_-VASc score [17–19]. The CCI, DCSI, CHADS_2_ score and CHA_2_DS_2_-VASc score were measured based on all observed conditions occurring at or prior to index.

### Angioedema outcome

We examined the safety outcome of angioedema, which was identified from diagnoses recorded on inpatient and emergency room healthcare claim records. Patients with a recorded diagnosis for angioedema at or within any time prior to index were excluded from the study.

### Time‐at‐risk

The time-at-risk window was defined based on the intention-to-treat principle, and patients were followed from day 1 post-index to the earliest of July 31, 2019, end of continuous exposure to treatment based on a conservative persistence window allowing for a maximum of 30 days between drug exposures, or end of continuous observation (i.e., the end of contiguous coverage or death) in the database [20]. Analyses were limited to patients with a minimum time-at-risk of 1 day.

### Large‐scale propensity score matching

Candidate covariates were defined as all aforementioned patient demographic and clinical characteristics, and heuristic feature selection was used to identify candidate covariates with a frequency greater than 0.1 %. We developed propensity models using LASSO regression with 10-fold cross-validation for hyperparameter tuning including all candidate covariates identified through heuristic feature selection, and propensity scores were calculated using the propensity model [8]. New users of ACEI and thiazide or thiazide-like diuretic monotherapy were matched at a 1:1 ratio using greedy matching enforcing a caliper of 0.10 and 0.20 of the pooled standard deviation of the logit of propensity scores in two separate analyses. To facilitate comparisons between LS-CM and LS-PSM, we defined matching covariates as candidate covariates with non-zero beta coefficients in the propensity model. LS-PSM was conducted in R version 3.6.3 using the Health Analytics Data-to-Evidence Suite (HADES) [21].

### Large‐scale cardinality matching

Matching covariates – covariates used in LS-CM - were empirically selected; matching covariates included candidate covariates with non-zero beta coefficients in the propensity model developed during LS-PSM. As such, matching covariates were identical between LS-PSM and LS-CM with one notable exception: due to memory constraints associated with LS-CM, in analyses of the full study population, heuristic feature selection was used to identify candidate covariates with a frequency threshold of 2 % instead of 0.1 %. Specifically, while LS-CM failed to converge to a matched sample due to insufficient memory while attempting to match on approximately 220 million data points (172,117 patients and 1,237 matching covariates), a matched sample was identified by LS-CM from a dataset containing over 120 million data points (172,117 patients and 717 matching covariates).

CM utilizes advancements in optimization algorithms to solve for the largest sample size meeting prespecified balance criteria (e.g., maximum standardized mean difference [SMD] of matching covariates) [4]. We performed LS-CM using the following prespecified balance criteria in four separate analyses: exact marginal distributional balance (i.e., fine balance; SMD = 0) and maximum SMD of 0.01, 0.05 and 0.10 of matching covariates between study groups.

All analyses were performed using an Amazon Web Services (AWS) Virtual Private Cloud (VPCx) m4.4xlarge Elastic Compute Cloud (EC2) instance. This instance included 16 2.3 GHz Intel® Xeon® vCPUs, 64 GiB of memory and a dedicated Elastic Block Storage (EBS) bandwidth of 2000 Mbps. Furthermore, all analyses were performed in R version 3.6.3 using Gurobi™ solver and the designmatch library.

### Evaluation of post‐match sample size

We evaluated patient retention in the matched samples subsequent to LS-PSM and LS-CM based on the average post-match sample size across all subsample draws within each sample group.

### Evaluation of post‐match covariate balance

The performance of LS-PSM and LS-CM were compared in terms of post-match covariate balance. The level of balance achieved indirectly and directly through matching was assessed based on candidate and matching covariate balance, respectively. SMDs, as defined by Rosenbaum & Rubin, were used to assess the post-match balance of candidate and matching covariates; specifically,

$$SMD = (\overline{x}_{treatment} - \overline{x}_{comparator}) / s_{p}$$

where x̄_treatment_ and x̄_comparator_ represent the post-match covariate mean of treatment and comparator group, respectively, and s_p_ represents the pre-match covariate pooled standard deviation [22]. An absolute SMD less than 0.10 was considered balanced. For each subsample group, the SMD of all candidate and matching covariates across all subsample draws were pooled such that covariate balance was assessed across all subsample draws considered jointly.

### Evaluation of post‐match residual confounding

Residual study bias due to unmeasured potential confounders and systematic error may still exist subsequent to LS-PSM or LS-CM [11, 12]. To quantify the magnitude of residual study bias, we included a total of 105 negative control outcomes in our experiment believed to be associated with neither ACEIs nor thiazide or thiazide-like diuretics, which, therefore, have a true hazard ratio equal to 1 [11]. These negative control outcomes were identified through a data-rich algorithm and manual clinical review (see Supplemental Appendix C for a list of negative control outcomes used in the current study) [23]. Hazard ratios were estimated for negative control outcomes using unconditional Cox proportional hazards models. Due to the increasingly limited number of negative control outcomes observed at smaller sample sizes, hazard ratios were estimated from all post-match observations pooled across all subsample draws within each subsample group.

Comparing the estimated hazard ratios of the negative control outcome experiments to the ground truth (of no effect) provides insight into residual study bias. We assume the observed log hazard ratio (${\hat{\uptheta}}_{\mathrm{i}}$) depends on the log of the true effect size (θ_i_), which is assumed to be 0, plus a systematic error component (β_i_), and let τ_i_ denote the standard error corresponding to θ_i_. Furthermore, we assume β_i_ to be distributed following a normal distribution with parameters μ and σ [2], which we estimate using the observed estimates (i.e., ${\hat{\uptheta}}_{\mathrm{i}}$) of negative control outcomes [24]. In summary, we develop a systematic error model based on the difference in the observed and expected effect estimates of negative control outcomes not attributable to random error based on the following assumptions:

$$\hat{\theta}_i \sim N (\theta_i + \beta_i, \tau_i^{2})$$

and

$$\beta_{i} \sim N (\mathrm{\mu}, \sigma^{2})$$

To summarize the systematic error component of negative control outcomes into a single measure we computed the expected systematic error (ESE), defined as the expected absolute systematic error based on the estimated null distribution parameters:

$$ESE = E(|\beta_{i}|)$$

It follows that a higher ESE may be attributable to increased systematic bias and, therefore, post-match residual bias. Conversely, a lower post-match ESE may be attributable to decreased systematic error and residual bias. Given a finite number of negative control outcomes and uncertainty in estimated hazard ratios due to limited sample size, the distribution parameters and, therefore, the ESE come with uncertainty, which we quantified using Markov-Chain Monte Carlo and expressed as 95 % credible intervals.

### Analyses of angioedema outcome

Unconditional Cox proportional hazards models were used to compare the safety outcome of angioedema between study groups within the full study population; insufficient occurrences of the outcome were observed to perform analyses within subsample groups. All hazard ratio (HR) estimates, 95 % confidence intervals (CI) and p-values were calibrated to incorporate the uncertainty expressed in the empirical null distribution of negative control outcomes as described by Schuemie et al. [11, 24]. Briefly, the aforementioned statistics were calibrated based on the systematic error model developed from negative control outcome experiments as previously discussed. We considered a two-sided calibrated p-value < 0.05 to be statistically significant. For reference, we further examined uncalibrated effect estimates.

## Results

### Post‐match sample size

The study inclusion criteria were met by 172,117 patients in the CCAE database, of which 129,078 (75.0 %) and 43,039 (25.0 %) were new users of ACEI and thiazide or thiazide-like monotherapy, respectively. Each subsample draw for the 10 %, 1 and 0.5 % subsample groups included 17,210 (ACEI: 12,907; thiazide or thiazide-like diuretic: 4,303), 1,720 (ACEI: 1290; thiazide or thiazide-like diuretic: 430) and 860 (ACEI: 645; thiazide or thiazide-like diuretic: 215) patients, respectively.

**Fig. 1:**
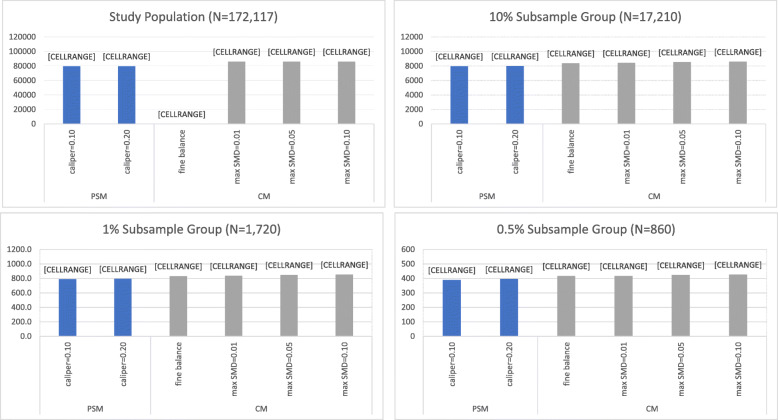
Average sample size after large-scale propensity score matching (PSM) and cardinality matching (CM). Fine balance: exact marginal distributional balance. * Failed to converge to a matched sample

The average post-match sample size across all analyses is shown in Fig. 1. In the full study population, LS-CM failed to converge to an optimal solution while requiring fine balance of matching covariates but was able to match every patient in the thiazide or thiazide-like diuretic group to a patient in the ACEI group (matched sample size = 86,078) at all other prespecified balance criteria. The use of more stringent balance criteria and a tighter caliper was associated with a slight reduction in post-match patient retention in LS-CM and LS-PSM, respectively, within subsample group analyses. With the exception of LS-CM requiring fine balance of matching covariates, LS-CM was associated with greater sample size retention as compared to LS-PSM.

### Post‐match matching covariate balance

In the full study population, 1,237 matching covariates were identified by LASSO regression for analyses using LS-PSM. Due to memory constraints associated with LS-CM at larger sample sizes, the frequency threshold of heuristic feature selection used to limit candidate covariates considered during LASSO regression was increased from 0.1 to 2 % for analyses within the full study population using LS-CM, which led to the identification of 717 matching covariates. An average of 210.6 (standard deviation [sd] = 43.7), 42.0 (sd = 19.6) and 23.2 (sd = 9.3) matching covariates were identified by LASSO regression across all subsample draws within the 10 %, 1 and 0.5 % subsample groups, respectively.

**Fig. 2:**
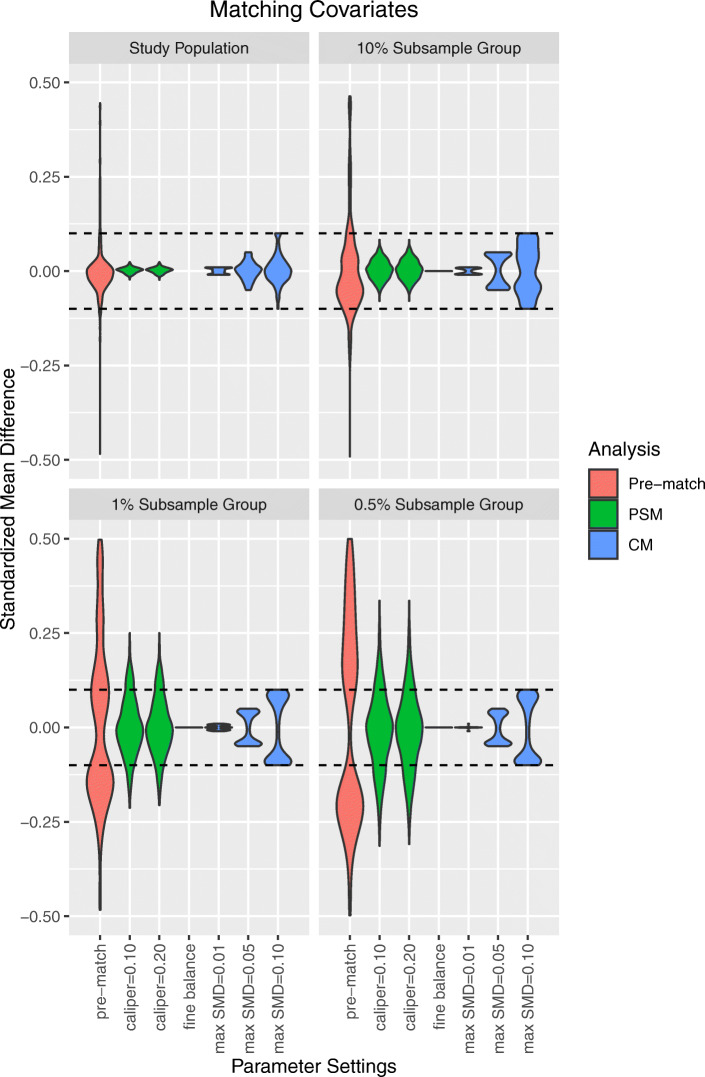
Matching covariate standardized mean differences after large-scale propensity score matching (PSM) and cardinality matching (CM). Fine balance: exact marginal distributional balance. Violin plots illustrate kernel probability density; the width of the shaded area represents the proportion of observations with the corresponding y-axis value

Figure 2 depicts the SMD of matching covariates across all analyses, and summary statistics on the average absolute SMD of matching covariates are available in Supplemental Appendix D. As evidenced by absolute SMDs greater than 0.10, significant matching covariate imbalance existed prior to matching. After LS-CM, no imbalanced matching covariates were observed within either the full study population or any subsample group. At small sample sizes, the distribution of matching covariate SMDs were skewed towards the prespecified balance criteria. Furthermore, more stringent prespecified balance criteria were associated with a reduction in the average SMD of matching covariates; and LS-CM requiring exact marginal distributional balance achieved perfect balance (e.g., SMD = 0, sd = 0) of matching covariates. While LS-PSM achieved balance across all matching covariates within the full study population and 10 % subsample group, the average frequency and proportion of post-match matching covariate imbalance within the 1 and 0.5 % subsample groups were: caliper = 0.10, 8.2 (19.6 %) and 7.6 (32.7 %), respectively; and caliper = 0.20, 8.4 (19.9 %) and 7.5 (32.5 %), respectively.

### Post‐match candidate covariate balance

A total of 50,391 candidate covariates were observed in the full study population. Due to a decrease in sample size, fewer candidate covariates were observed among subsample groups. The average number of candidate covariates observed within the 10 %, 1 and 0.5 % subsample groups was 26,696.8 (sd = 467.4), 11,644.2 (sd = 441.9) and 8,581.1 (sd = 436.1), respectively.

**Fig. 3:**
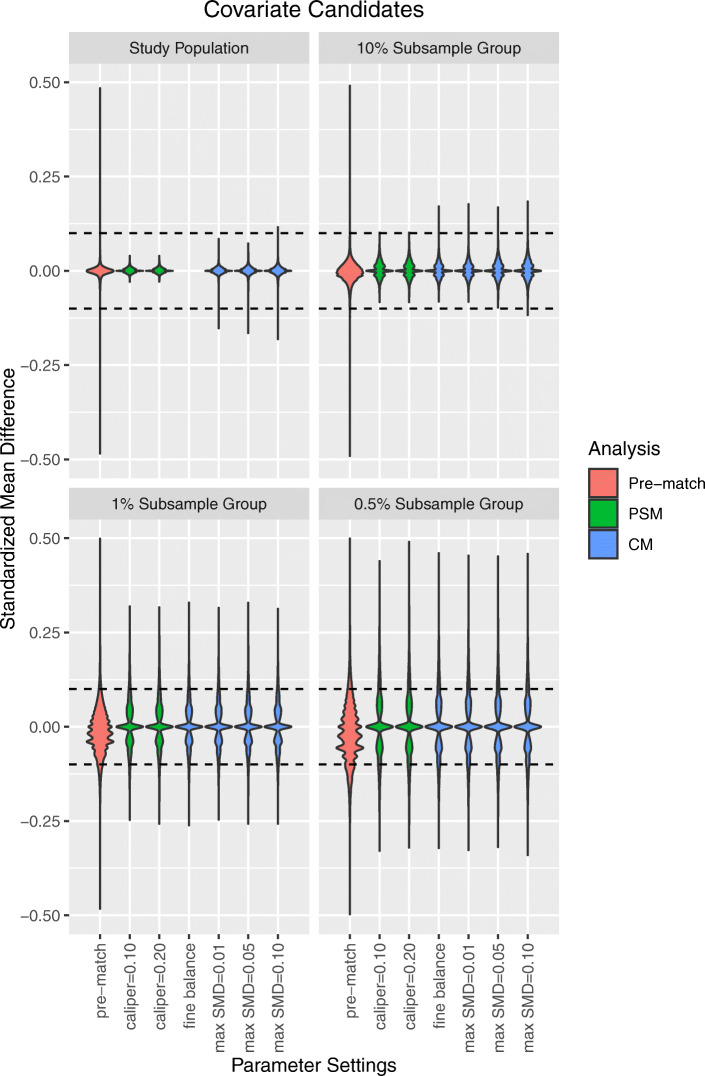
Candidate covariate standardized mean differences after large-scale propensity score matching (PSM) and cardinality matching (CM). Fine balance: exact marginal distributional balance. Violin plots illustrate kernel probability density; the width of the shaded area represents the proportion of observations with the corresponding y-axis value

The SMD of candidate covariates before matching and across all analyses is shown in Fig. 3. Overall, post-match candidate covariate imbalance was negatively correlated with sample size, and, likewise, covariate overlap was increasingly limited at smaller sample sizes in the pre-match sample. In the full study population, no imbalanced covariates were observed following LS-PSM (see Supplemental Appendix E). Similarly, LS-PSM was associated with a small, albeit non-significant, improvement in the average SMD of candidate covariates in the full study population as compared to LS-CM (see Supplemental Appendix F). Nevertheless, comparable improvements in candidate covariate balance were achieved by both matching techniques within each subsample group.

### Post‐match residual confounding

**Fig. 4:**
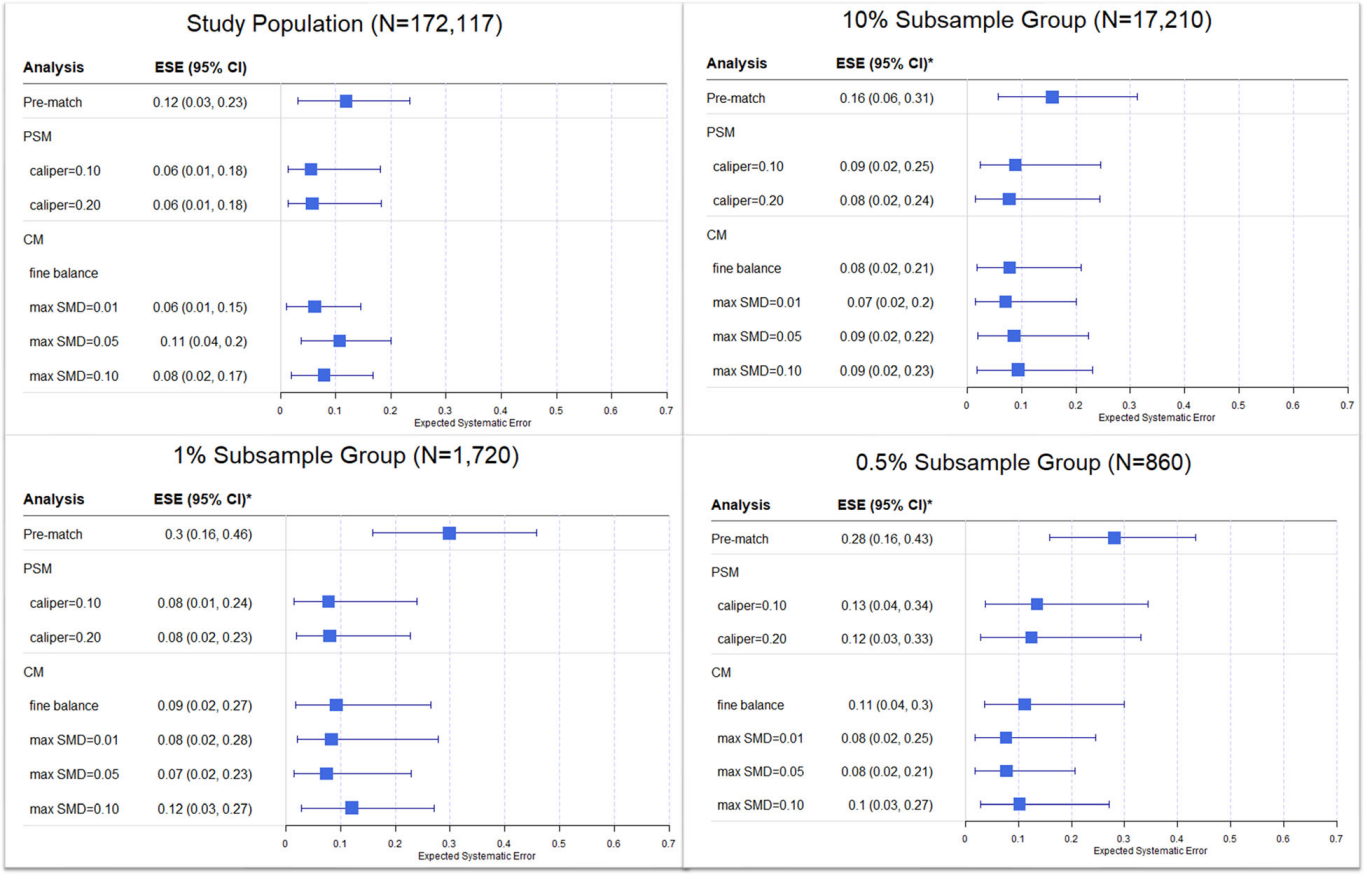
Expected systematic error of negative controls after large-scale propensity score matching (PSM) and cardinality matching (CM). ESE: expected systematic error; fine balance: exact marginal distributional balance

The expected systematic error (ESE) prior to matching and subsequent to LS-PSM and LS-CM within the full study population and each subsample group is shown in Fig. 4. Overall, ESE was negatively correlated with sample size albeit non-significantly. As compared to the pre-match sample, both matching techniques were associated with similar reductions in ESE (e.g., 0.5 % subsample group: pre-match, ESE = 0.28 [95 % CI: (0.16, 0.43)]; LS-PSM with caliper = 0.10, ESE = 0.13 [95 % CI: (0.04, 0.34)]; and LS-CM with maximum SMD = 0.01, ESE = 0.08 [95 % CI: (0.02, 0.25)]).

### Analyses of angioedema outcome

**Fig. 5:**
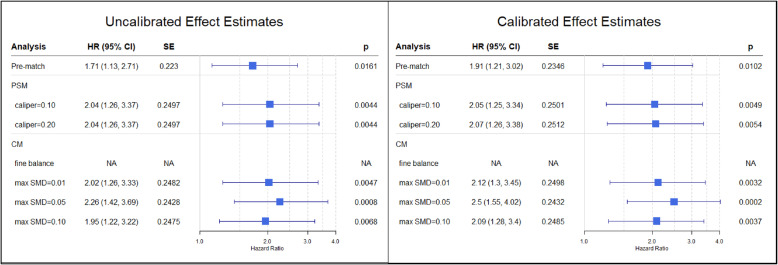
Hazard ratio for angioedema between new users of ACEI vs. thiazide or thiazide-like diuretic monotherapy^a^. ACEI: angiotensin-converting enzyme inhibitor; HR: hazard ratio; CI: confidence interval; SE: standard error of the natural log of the hazard ratio; PSM: lage-scale propensity score matching; CM: large-scale cardinality matching.^a^ Uncalibrated effect estimates (left panel) and calibrated effect estimates (right panel) for analyses performed within the full study population (*N*=172,117). Calibrated hazard ratio, estimates, confidence intervals, standard errors and p-values based on the empirical null distribution of negative control outcomes

Results from analyses of the safety outcome of angioedema between new users of ACEI vs. thiazide and thiazide-like monotherapy within the full study population are presented in Fig. 5. As compared to thiazide or thiazide-like monotherapy, ACEI monotherapy was found to be associated with a significant increase in the risk of angioedema across all analyses (calibrated *p* < 0.05), and calibrated HR estimates did not significantly differ between LS-PSM and LS-CM. Furthermore, LS-CM was associated with a slight decrease in the standard error of calibrated HR estimates relative to LS-PSM. Similar trends were observed among uncalibrated effect estimates.

## Discussion

In this applied comparison of LS-PSM and LS-CM among new users of ACEI vs. thiazide and thiazide-like diuretic monotherapy, LS-CM found the largest matched sample meeting prespecified balance criteria. The current study proposed a framework for the empirical selection of matching covariates for LS-CM and assessed the performance of both matching techniques at progressively smaller sample sizes with increasingly limited covariate overlap. While both matching techniques achieved similar candidate covariate balance, LS-CM was associated with improved matching covariate balance in analyses with smaller sample sizes. Furthermore, LS-CM was associated with improved patient retention as compared to LS-PSM translating to slight improvements in the precision of effect estimates. Finally, LS-CM and LS-PSM were associated with similar improvements in residual confounding, which was assessed based on the ESE of negative control outcome experiments.

Prior literature comparing CM and PSM is limited. In a study examining the impact of earthquakes on electoral outcomes in Chile, Visconti et al. describe the performance of both matching techniques. Before matching, the study included a total of 172 observations. As compared to PSM, CM was associated with a decrease in both post-match sample size (108 vs. 154) and, as evidenced by a SMD greater than 0.10, matching covariate imbalance (0 vs. 13 out of 18 imbalanced matching covariates) [4]. Similarly, in a Monte Carlo simulation study, de los Angeles Resa and Zubizarreta found CM to systematically select the largest sample size meeting a set of prespecified balance criteria [10].

Consistent with prior literature, as evidenced by a SMD less than 0.10, LS-CM achieved balance for all matching covariates across all analyses. While LS-PSM achieved balance of all matching covariates in analyses with larger sample sizes (e.g., full study population and 10 % subsample groups), the matching technique was associated with substantial matching covariate imbalance in analyses with smaller sample sizes (e.g., the 1 and 0.5 % subsample groups). Furthermore, LS-CM was associated with improved sample retention across all analyses with the exception of fine balance within the study population, which failed to converge to an optimal solution, indicating that the achievement of prespecified balance criteria was not mutually exclusive to superior sample size retention. In the case that LS-CM fails to converge to a solution, we recommend the investigator consider loosening the prespecified balance criteria or implementing post-match statistical adjustments. Finally, in analyses of smaller sample sizes, the distribution of matching covariate SMDs were skewed towards the prespecified balance criteria, which supports the use of tighter prespecified balance criteria albeit the potential trade-off in patient retention should also be considered.

Both candidate covariate imbalance and ESE were negatively correlated with sample size. As compared to the pre-match sample, improvements in candidate covariate balance were achieved with either matching technique; however, LS-PSM achieved better candidate covariate balance in analyses with larger sample sizes. That being said, it is important to note that fewer matching covariates were used with LS-CM as compared to LS-PSM (717 vs. 1,237) in analyses within the full study population due to memory limitations associated with LS-CM. Similarly, reductions in residual confounding as indicated by the ESE of negative control outcome experiments were comparable between LS-PSM and LS-CM. A gradual, albeit non-significant, increase in ESE was observed with LS-PSM at progressively smaller sample sizes potentially due to limited effective degrees of freedom. These findings may indicate both matching techniques are comparable in reducing residual confounding stemming from imbalances in unmeasured or otherwise unadjusted covariates.

Calibrated hazard ratio estimates were similar in direction and magnitude across all analyses within the study population indicating ACEI monotherapy was associated with a significant increase in the risk of angioedema as compared to thiazide or thiazide-like monotherapy. However, as compared to LS-PSM, LS-CM was associated with a slight reduction in the standard error of estimates. Similar trends were observed among uncalibrated analyses. These findings are consistent with those reported de los Angeles Resa and Zubizarreta, which reported lower root-mean square errors associated with CM [10]. The increased precision of effect estimates may be due to the improved sample retention observed with LS-CM.

## Limitations

The current study was subject to limitations. First, due to memory constraints, the identification of matching covariates through LASSO regression within the full study population was limited to covariates with a minimum frequency of 2 % for LS-CM and 0.1 % for LS-PSM. As such, the performance of LS-CM as compared to LS-PSM in addressing potential confounding within studies of large sample sizes may have been underestimated. Nevertheless, this highlights practical limitations of CM in large-scale studies associated with limitations in computing power; LS-CM failed to converge due to memory constraints using a dataset containing approximately 220 million data points (172,117 observations and 1,237 matching covariates) but successfully converged using a dataset containing approximately 120 million data points (172,117 observations and 717 matching covariates). These practical limitations may be overcome with access to more powerful computing resources.

Second, the use of negative control experiments limited analyses to subsample groups with a pre-match sample size sufficient to ensure the observation of negative control outcomes after matching. The current study addressed this limitation by pooling negative control outcome effect estimates across multiple sample draws, enabling the performance of negative control outcome experiments within subsample group containing as few as 860 patients prior to matching. Nevertheless, simulation studies may be necessary to explore residual confounding at even smaller sample sizes.

Finally, study findings may not be generalizable to other healthcare datasets. While the current study compared the performance of LS-PSM and LS-CM at progressively smaller sample sizes with increasingly limited covariate overlap, additional simulation studies may be warranted to increase the generalizability of results.

## Conclusions

The current study compared the performance of LS-PSM and LS-CM in terms of post-match sample size, covariate balance and residual confounding. LS-CM found the largest matched sample meeting prespecified balance criteria thereby achieving superior sample retention and, in analyses at smaller sample sizes with increasingly limited covariate overlap, improved matching covariate balance as compared with LS-PSM. Candidate covariate balance and residual bias were comparable between matching techniques. These findings support LS-CM as an alternative to LS-PSM for causal inference in observational research with small sample sizes where limited covariate overlap may result in poor matching covariate balance or limited patient retention. Further research is necessary to compare the performance of PSM and CM in studies where empirical covariate selection may not be possible due to limited sample size or availability of data.

## Supplementary Information

**Additional file 1.**

## Data Availability

The datasets generated and analyzed during the current study are not publicly available as they were obtained from IBM under a proprietary data use agreement but are available from the corresponding author on reasonable request. The code used to generate and analyze the datasets for the current study are available in the github, https://github.com/ohdsi-studies/EvaluatingCardinalityMatching.

## Abbreviations

AMA: American Heart Association
ACEI: Angiotensin-converting enzyme inhibitor
AHA: American Heart Association
ATC: Average Treatment Effect on the Controls
ATE: Average Treatment Effect
CCAE: IBM® MarketScan® Commercial Claims and Encounters Database
CCI: Charlson Comorbidity Index
CDM: Common Data Model
CI: Confidence interval
CM: Cardinality matching
DCSI: Diabetes Complications Severity Index
ESE: Expected systematic error
HADES: Health Analytics Data-to-Evidence Suite
HR: Hazard Ratio
LEGEND-HTN: Large-Scale Evidence Generation and Evaluation across a Network of Databases hypertension
LS-CM: Large-scale cardinality matching
LS-PSM: Large-scale propensity score matching
MCMC: Markov Chain Monte Carlo
OHDSI: Observational Health and Data Sciences and Informatics
OMOP: Observational Medical Outcomes Partnership
PSM: Propensity score matching
sd: Standard deviation
SMD: Standardized mean difference
