# Correction to: Applied comparison of large-scale propensity score matching and cardinality matching for causal inference in observational research

**DOI:** 10.1186/s12874-021-01365-z

**Published:** 2021-08-21

**Authors:** Stephen P. Fortin, Stephen S. Johnston, Martijn J. Schuemie

**Affiliations:** 1 Janssen R&D, LLC, Raritan, NJ USA; 2 Johnson & Johnson, New Brunswick, NJ USA

**Correction to: BMC Med Res Methodol 21, 109 (2021)**

**https://doi.org/10.1186/s12874-021-01282-1**

Following publication of the original article [1], the authors noticed an error in Fig. 2, and some cropping of the text in Fig. 3. Presented here are the updated Figs. 2 and 3. The original article has been updated.

**Fig. 2:**
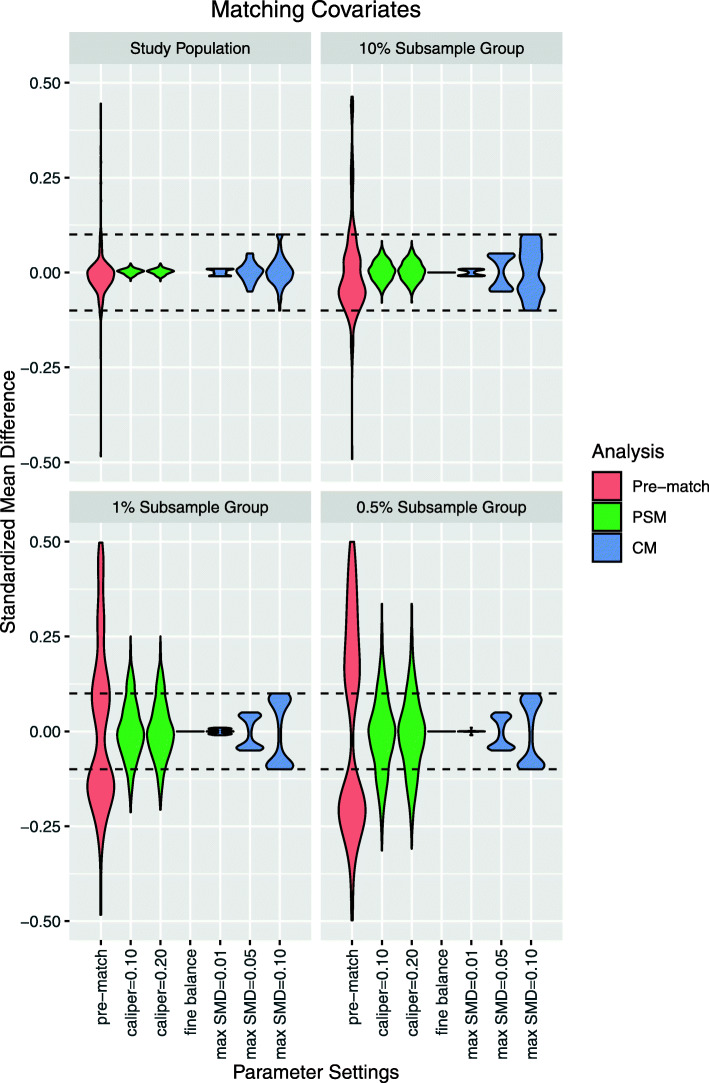
Matching covariate standardized mean differences after large-scale propensity score matching (PSM) and cardinality matching (CM). Fine balance: exact marginal distributional balance. Violin plots illustrate kernel probability density; the width of the shaded area represents the proportion of observations with the corresponding y-axis value

**Fig. 3:**
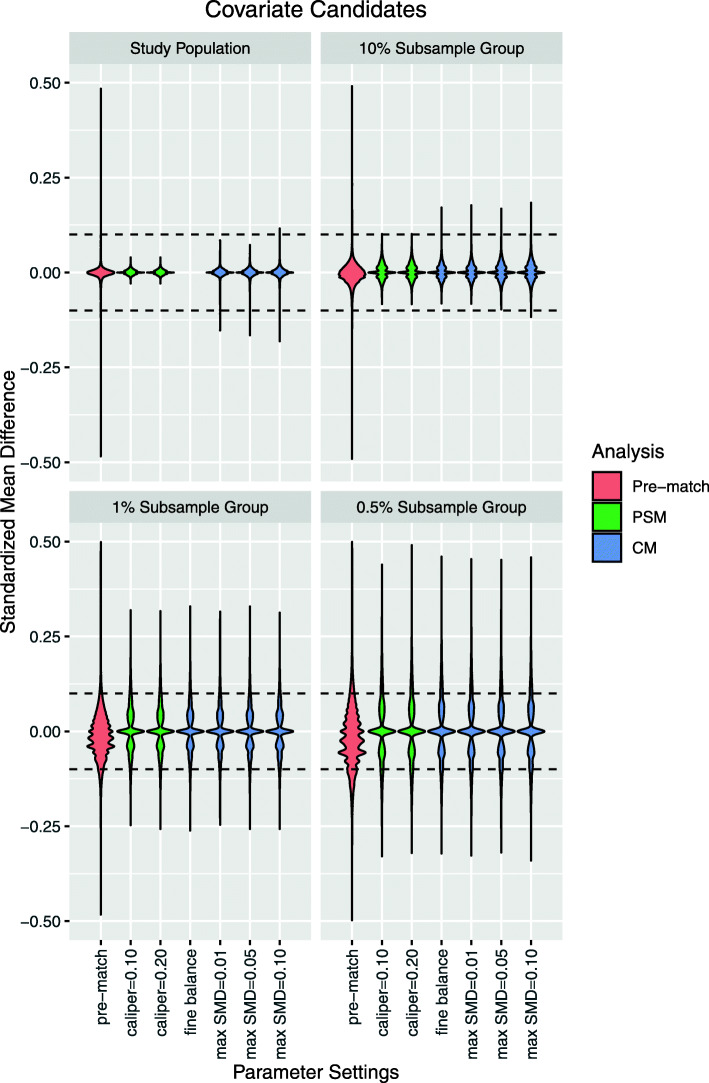
Candidate covariate standardized mean differences after large-scale propensity score matching (PSM) and cardinality matching (CM). Fine balance: exact marginal distributional balance. Violin plots illustrate kernel probability density; the width of the shaded area represents the proportion of observations with the corresponding y-axis value
