# Supplementary material for: Applied comparison of large‐scale propensity score matching and cardinality matching for causal inference in observational research
Source: BMC Med Res Methodol. 2021 May 24;21:109. doi: 10.1186/s12874-021-01282-1 (PMC8146256; doi:10.1186/s12874-021-01282-1)
Supplement: Supplementary file 1 — Additional file 1. [file 12874_2021_1282_MOESM1_ESM.docx]

**Supplemental Appendix A**. List of codes used to query the database

| Concept Set | Concept ID | Concept Description | Domain | Vocabulary |
| --- | --- | --- | --- | --- |
| Inpatient or ER visit | 262 | Emergency Room and Inpatient Visit | Visit | Visit |
|  | 9203 | Emergency Room Visit | Visit | Visit |
|  | 9201 | Inpatient Visit | Visit | Visit |
| Hypertensive disorder | 316866 | Hypertensive disorder | Condition | SNOMED |
| Angioedema | 432791 | Angioedema | Condition | SNOMED |
| Hypertension drugs | 1319998 | Acebutolol | Drug | RxNorm |
|  | 1317967 | aliskiren | Drug | RxNorm |
|  | 991382 | Amiloride | Drug | RxNorm |
|  | 1332418 | Amlodipine | Drug | RxNorm |
|  | 1314002 | Atenolol | Drug | RxNorm |
|  | 40235485 | azilsartan | Drug | RxNorm |
|  | 1335471 | benazepril | Drug | RxNorm |
|  | 1322081 | Betaxolol | Drug | RxNorm |
|  | 1338005 | Bisoprolol | Drug | RxNorm |
|  | 932745 | Bumetanide | Drug | RxNorm |
|  | 1351557 | candesartan | Drug | RxNorm |
|  | 1340128 | Captopril | Drug | RxNorm |
|  | 1346823 | carvedilol | Drug | RxNorm |
|  | 1395058 | Chlorthalidone | Drug | RxNorm |
|  | 1398937 | Clonidine | Drug | RxNorm |
|  | 1328165 | Diltiazem | Drug | RxNorm |
|  | 1363053 | Doxazosin | Drug | RxNorm |
|  | 1341927 | Enalapril | Drug | RxNorm |
|  | 1309799 | eplerenone | Drug | RxNorm |
|  | 1346686 | eprosartan | Drug | RxNorm |
|  | 1353776 | Felodipine | Drug | RxNorm |
|  | 1363749 | Fosinopril | Drug | RxNorm |
|  | 956874 | Furosemide | Drug | RxNorm |
|  | 1344965 | Guanfacine | Drug | RxNorm |
|  | 1373928 | Hydralazine | Drug | RxNorm |
|  | 974166 | Hydrochlorothiazide | Drug | RxNorm |
|  | 978555 | Indapamide | Drug | RxNorm |
|  | 1347384 | irbesartan | Drug | RxNorm |
|  | 1326012 | Isradipine | Drug | RxNorm |
|  | 1386957 | Labetalol | Drug | RxNorm |
|  | 1308216 | Lisinopril | Drug | RxNorm |
|  | 1367500 | Losartan | Drug | RxNorm |
|  | 1305447 | Methyldopa | Drug | RxNorm |
|  | 907013 | Metolazone | Drug | RxNorm |
|  | 1307046 | Metoprolol | Drug | RxNorm |
|  | 1309068 | Minoxidil | Drug | RxNorm |
|  | 1310756 | moexipril | Drug | RxNorm |
|  | 1313200 | Nadolol | Drug | RxNorm |
|  | 1314577 | nebivolol | Drug | RxNorm |
|  | 1318137 | Nicardipine | Drug | RxNorm |
|  | 1318853 | Nifedipine | Drug | RxNorm |
|  | 1319880 | Nisoldipine | Drug | RxNorm |
|  | 40226742 | olmesartan | Drug | RxNorm |
|  | 1327978 | Penbutolol | Drug | RxNorm |
|  | 1373225 | Perindopril | Drug | RxNorm |
|  | 1345858 | Pindolol | Drug | RxNorm |
|  | 1350489 | Prazosin | Drug | RxNorm |
|  | 1353766 | Propranolol | Drug | RxNorm |
|  | 1331235 | quinapril | Drug | RxNorm |
|  | 1334456 | Ramipril | Drug | RxNorm |
|  | 970250 | Spironolactone | Drug | RxNorm |
|  | 1317640 | telmisartan | Drug | RxNorm |
|  | 1341238 | Terazosin | Drug | RxNorm |
|  | 942350 | torsemide | Drug | RxNorm |
|  | 1342439 | trandolapril | Drug | RxNorm |
|  | 904542 | Triamterene | Drug | RxNorm |
|  | 1308842 | valsartan | Drug | RxNorm |
|  | 1307863 | Verapamil | Drug | RxNorm |
| Thiazide or thiazide-like diuretics | 1395058 | Chlorthalidone | Drug | RxNorm |
|  | 974166 | Hydrochlorothiazide | Drug | RxNorm |
|  | 978555 | Indapamide | Drug | RxNorm |
|  | 907013 | Metolazone | Drug | RxNorm |
| Angiotensin converting enzyme inhibitors | 1335471 | benazepril | Drug | RxNorm |
|  | 1340128 | Captopril | Drug | RxNorm |
|  | 1341927 | Enalapril | Drug | RxNorm |
|  | 1363749 | Fosinopril | Drug | RxNorm |
|  | 1308216 | Lisinopril | Drug | RxNorm |
|  | 1310756 | moexipril | Drug | RxNorm |
|  | 1373225 | Perindopril | Drug | RxNorm |
|  | 1331235 | quinapril | Drug | RxNorm |
|  | 1334456 | Ramipril | Drug | RxNorm |
|  | 1342439 | trandolapril | Drug | RxNorm |

* Concept sets include all descendants of listed concept IDs

**Supplemental Appendix B.** Patient attrition table.

| Step | Description | Patient attrition, n (%) | | |
| --- | --- | --- | --- | --- |
|  |  | Overall | Thiazide Group | ACEI Group |
| 1 | Drug exposure to ACEI or thiazide or thiazide-like diuretic for the first time in patient's history. Limited to drug exposures occurring between October 1, 2014 and January 1, 2017 with a minimum of 365 days of prior continuous observation. | 793,946 (100%) | 371,243 (100%) | 422,703 (100%) |
| 2 | Diagnosis of hypertensive disorder present at or within 365 days prior to index. | 624,495 (78.7%) | 293,880 (79.2%) | 330,615 (78.2%) |
| 3 | No drug exposures to any other active ingredient listed within the five primary drug classes for the treatment of hypertension in the ACC/AHA guidelines at any time prior to or within 7 days post-index. | 172,696 (21.8%) | 43,182 (11.6%) | 129,514 (30.6%) |
| 4 | No prior angioedema outcome. | 172,564 (21.7%) | 43,133 (11.6%) | 129,431 (30.6%) |
| 5 | Minimum time-at-risk of 1 day. | 172,117 (21.7%) | 43,039 (11.6%) | 129,078 (30.5%) |

ACEI: angiotensin-converting enzyme inhibitor

**Supplemental Appendix C**. List of negative control outcomes used in the current study

| Concept Id | Concept Description | Domain | Vocabulary |
| --- | --- | --- | --- |
| 46286594 | Problem related to lifestyle | Condition | SNOMED |
| 46269889 | Complication due to Crohn's disease | Condition | SNOMED |
| 45757370 | Disproportion of reconstructed breast | Condition | SNOMED |
| 44783954 | Acid reflux | Condition | SNOMED |
| 42873170 | Dependence on supplemental oxygen | Condition | SNOMED |
| 40481632 | Ganglion cyst | Condition | SNOMED |
| 40480893 | Nonspecific tuberculin test reaction | Condition | SNOMED |
| 36717682 | Somatic dysfunction of sacral region | Condition | SNOMED |
| 36717115 | Somatic dysfunction of head region | Condition | SNOMED |
| 36713926 | Somatic dysfunction of thoracic region | Condition | SNOMED |
| 36713918 | Somatic dysfunction of lumbar region | Condition | SNOMED |
| 4344500 | Impingement syndrome of shoulder region | Condition | SNOMED |
| 4248728 | Snoring | Condition | SNOMED |
| 4241530 | Asymptomatic human immunodeficiency virus infection | Condition | SNOMED |
| 4231770 | Hereditary thrombophilia | Condition | SNOMED |
| 4213540 | Cervical somatic dysfunction | Condition | SNOMED |
| 4209423 | Nicotine dependence | Condition | SNOMED |
| 4202045 | Postviral fatigue syndrome | Condition | SNOMED |
| 4201717 | Ileostomy present | Condition | SNOMED |
| 4201390 | Colostomy present | Condition | SNOMED |
| 4170770 | Epidermoid cyst | Condition | SNOMED |
| 4166231 | Genetic predisposition | Condition | SNOMED |
| 4119307 | Neurogenic claudication | Condition | SNOMED |
| 4115402 | Difficulty sleeping | Condition | SNOMED |
| 4115367 | Wrist joint pain | Condition | SNOMED |
| 4103703 | Melena | Condition | SNOMED |
| 4103640 | Amputated foot | Condition | SNOMED |
| 4092896 | Feces contents abnormal | Condition | SNOMED |
| 4092879 | Absent kidney | Condition | SNOMED |
| 4091513 | Passing flatus | Condition | SNOMED |
| 4088290 | Absence of breast | Condition | SNOMED |
| 4083487 | Macular drusen | Condition | SNOMED |
| 4012934 | Homocystinuria | Condition | SNOMED |
| 4012570 | High risk sexual behavior | Condition | SNOMED |
| 4002818 | Spasm of back muscles | Condition | SNOMED |
| 444132 | Injury of knee | Condition | SNOMED |
| 443172 | Splinter of face, without major open wound | Condition | SNOMED |
| 442306 | Adjustment disorder with depressed mood | Condition | SNOMED |
| 441788 | Human papilloma virus infection | Condition | SNOMED |
| 441277 | Mixed receptive-expressive language disorder | Condition | SNOMED |
| 440704 | Chronic pain syndrome | Condition | SNOMED |
| 440329 | Herpes zoster without complication | Condition | SNOMED |
| 440193 | Wristdrop | Condition | SNOMED |
| 440021 | Herpes simplex without complication | Condition | SNOMED |
| 439935 | Abnormal posture | Condition | SNOMED |
| 439790 | Psychalgia | Condition | SNOMED |
| 439776 | Autism spectrum disorder | Condition | SNOMED |
| 438329 | Late effect of motor vehicle accident | Condition | SNOMED |
| 438130 | Opioid abuse | Condition | SNOMED |
| 438120 | Opioid dependence | Condition | SNOMED |
| 437969 | Talipes planus | Condition | SNOMED |
| 437264 | Tobacco dependence syndrome | Condition | SNOMED |
| 437092 | Physiological development failure | Condition | SNOMED |
| 436785 | Spinal stenosis in cervical region | Condition | SNOMED |
| 436409 | Abnormal pupil | Condition | SNOMED |
| 436373 | Developmental speech disorder | Condition | SNOMED |
| 434626 | Borderline personality disorder | Condition | SNOMED |
| 434327 | Cannabis abuse | Condition | SNOMED |
| 434203 | Late effect of contusion | Condition | SNOMED |
| 434165 | Abnormal cervical smear | Condition | SNOMED |
| 433577 | Hammer toe | Condition | SNOMED |
| 433527 | Endometriosis | Condition | SNOMED |
| 433111 | Effects of hunger | Condition | SNOMED |
| 432593 | Kwashiorkor | Condition | SNOMED |
| 432303 | Cocaine abuse | Condition | SNOMED |
| 380706 | Regular astigmatism | Condition | SNOMED |
| 378427 | Tear film insufficiency | Condition | SNOMED |
| 378424 | Astigmatism | Condition | SNOMED |
| 377572 | Noise effects on inner ear | Condition | SNOMED |
| 376707 | Acute conjunctivitis | Condition | SNOMED |
| 376382 | Tension-type headache | Condition | SNOMED |
| 374375 | Impacted cerumen | Condition | SNOMED |
| 373478 | Presbyopia | Condition | SNOMED |
| 259995 | Foreign body in orifice | Condition | SNOMED |
| 201606 | Crohn's disease | Condition | SNOMED |
| 199192 | Abrasion and/or friction burn of trunk without infection | Condition | SNOMED |
| 196168 | Irregular periods | Condition | SNOMED |
| 195873 | Leukorrhea | Condition | SNOMED |
| 195590 | Urethral stricture | Condition | SNOMED |
| 194083 | Vaginitis and vulvovaginitis | Condition | SNOMED |
| 141932 | Senile hyperkeratosis | Condition | SNOMED |
| 140842 | Changes in skin texture | Condition | SNOMED |
| 140648 | Onychomycosis due to dermatophyte | Condition | SNOMED |
| 140641 | Verruca vulgaris | Condition | SNOMED |
| 139099 | Ingrowing nail | Condition | SNOMED |
| 137951 | Acquired keratoderma | Condition | SNOMED |
| 136773 | Rosacea | Condition | SNOMED |
| 136368 | Non-toxic multinodular goiter | Condition | SNOMED |
| 134438 | Contact dermatitis | Condition | SNOMED |
| 133655 | Burn of forearm | Condition | SNOMED |
| 81634 | Ptotic breast | Condition | SNOMED |
| 81378 | Chondromalacia of patella | Condition | SNOMED |
| 81151 | Sprain of ankle | Condition | SNOMED |
| 80502 | Osteoporosis | Condition | SNOMED |
| 79864 | Hematuria syndrome | Condition | SNOMED |
| 78619 | Contusion of knee | Condition | SNOMED |
| 77965 | Acquired trigger finger | Condition | SNOMED |
| 76786 | Derangement of knee | Condition | SNOMED |
| 75911 | Acquired hallux valgus | Condition | SNOMED |
| 74464 | Idiopathic scoliosis AND/OR kyphoscoliosis | Condition | SNOMED |
| 73754 | Restless legs | Condition | SNOMED |
| 73560 | Calcaneal spur | Condition | SNOMED |
| 73241 | Anal and rectal polyp | Condition | SNOMED |
| 72748 | Strain of rotator cuff capsule | Condition | SNOMED |
| 72404 | Joint stiffness | Condition | SNOMED |

**Supplemental Appendix D**. Average matching covariate standardized mean differences after large-scale propensity score matching and cardinality matching

| Analysis | Study Population,  mean (sd) | Subsample Group, mean (sd) | | |
| --- | --- | --- | --- | --- |
|  |  | 10% | 1% | 0.5% |
| Pre-match | 0.041 (0.058) | 0.087 (0.09) | 0.183 (0.122) | 0.253 (0.121) |
| PSM |  |  |  |  |
| caliper=0.10 | 0.006 (0.005) | 0.021 (0.015) | 0.06 (0.047) | 0.079 (0.064) |
| caliper=0.20 | 0.006 (0.005) | 0.021 (0.015) | 0.06 (0.047) | 0.08 (0.064) |
| CM |  |  |  |  |
| fine balance | - | 0 (0) | 0 (0) | 0 (0) |
| max SMD=0.01 | 0.007 (0.003) | 0.007 (0.002) | 0.005 (0.003) | 0.001 (0.003) |
| max SMD=0.05 | 0.019 (0.014) | 0.037 (0.013) | 0.038 (0.012) | 0.035 (0.012) |
| max SMD=0.10 | 0.028 (0.026) | 0.055 (0.029) | 0.079 (0.022) | 0.076 (0.023) |

PSM: propensity score matching; CM: cardinality matching; fine balance: exact marginal distributional balance

**Supplementary Appendix E**. Average frequency of candidate covariate imbalance after large-scale propensity score matching and cardinality matching

| Analysis | Study Population, n (%)  (N=50,391)^a^ | Subsample Group, n (%) | | |
| --- | --- | --- | --- | --- |
|  |  | 10% (N=26,696.8)^b^ | 1% (N=11,664.2)^b^ | 0.5% (N=8,581.1)^b^ |
| Pre-match | 217 (0.4%) | 269.2 (1%) | 584.7 (5%) | 968.72 (11.3%) |
| PSM |  |  |  |  |
| caliper=0.10 | 0 (0%) | 0.4 (0%) | 577.86 (5%) | 856.63 (10%) |
| caliper=0.20 | 0 (0%) | 0.4 (0%) | 573.72 (4.9%) | 857.79 (10%) |
| CM |  |  |  |  |
| fine balance | - | 5.2 (0%) | 522.96 (4.5%) | 855.76 (10%) |
| max SMD=0.01 | 0 (0%) | 5.2 (0%) | 522.96 (4.5%) | 855.76 (10%) |
| max SMD=0.05 | 2 (0%) | 4.4 (0%) | 525 (4.5%) | 854.63 (10%) |
| max SMD=0.10 | 4 (0%) | 5.8 (0%) | 515.4 (4.4%) | 847.3 (9.9%) |

PSM: propensity score matching; CM: cardinality matching; fine balance: exact marginal distributional balance; candidate covariate imbalance defined as SMD >0.10

^a^ N: average number of candidate covariates identified within the study population and across all subsample draws within each subsample group

**Supplementary Appendix F**. Average candidate covariate standardized mean differences after large-scale propensity score matching and cardinality matching

| Analysis | Study Population,  mean (sd) | Subsample Group, mean (sd) | | |
| --- | --- | --- | --- | --- |
|  |  | 10% | 1% | 0.5% |
| Pre-match | 0.010 (0.020) | 0.021 (0.029) | 0.049 (0.046) | 0.065 (0.057) |
| PSM |  |  |  |  |
| caliper=0.10 | 0.005 (0.004) | 0.015 (0.013) | 0.045 (0.041) | 0.061 (0.057) |
| caliper=0.20 | 0.005 (0.004) | 0.015 (0.013) | 0.045 (0.041) | 0.061 (0.056) |
| CM |  |  |  |  |
| fine balance | - | 0.014 (0.013) | 0.043 (0.039) | 0.059 (0.055) |
| max SMD=0.01 | 0.006 (0.006) | 0.014 (0.013) | 0.043 (0.039) | 0.059 (0.055) |
| max SMD=0.05 | 0.007 (0.008) | 0.017 (0.015) | 0.043 (0.039) | 0.059 (0.055) |
| max SMD=0.10 | 0.008 (0.010) | 0.019 (0.017) | 0.044 (0.039) | 0.059 (0.054) |

PSM: propensity score matching; CM: cardinality matching; fine balance: exact marginal distributional balance
